# TagSeq: Malicious behavior discovery using dynamic analysis

**DOI:** 10.1371/journal.pone.0263644

**Published:** 2022-05-16

**Authors:** Yi-Ting Huang, Yeali S. Sun, Meng Chang Chen

**Affiliations:** 1 Institute of Information Science, Academia Sinica, Taipei, Taiwan; 2 Department of Information Management, National Taiwan University, Taipei, Taiwan; Politechnika Slaska, POLAND

## Abstract

In recent years, studies on malware analysis have noticeably increased in the cybersecurity community. Most recent studies concentrate on malware classification and detection or malicious patterns identification, but as to malware activity, it still relies heavily on manual analysis for high-level semantic descriptions. We develop a sequence-to-sequence (seq2seq) neural network, called TagSeq, to investigate a sequence of Windows API calls recorded from malware execution, and produce tags to label their malicious behavior. We propose embedding modules to transform Windows API function parameters, registry, filenames, and URLs into low-dimension vectors, while still preserving the closeness property. Moreover, we utilize an attention mechanism to capture the relations between generated tags and certain API invocation calls. Results show that the most possible malicious actions are identified by TagSeq. Examples and a case study demonstrate that the proposed embedding modules preserve semantic-physical relations and that the predicted tags reflect malicious intentions. We believe this work is suitable as a tool to help security analysts recognize malicious behavior and intent with easy-to-understand tags.

## 1 Introduction

Malware (also called malicious software), such as Trojan horses, computer viruses, Internet worms, and ransomware, is a major challenge in cybersecurity, since it can be used to disrupt network service, destroy software, steal sensitive data, or take control of a host. Therefore, malware analysis has been extensively studied on the host-based environment [1–3]. Anti-virus products are primarily concerned with identifying individual malware signatures to detect malware. However, more recently, with the development of obfuscation techniques and prevailing access to open source tools, it has been easy to create malware variants, greatly increasing the amount of malware. Thus, rather than identifying malware signatures, we have shifted our attention to analyze malware behavior. Malicious characteristics that can be detected can form the basis of malware detection. This approach will increase the effectiveness of malware detection and decrease the operational costs thereof.

To the best of our knowledge, there is no benchmark for malicious characteristics, because it is difficult to examine infected systems, system logs, and malware binaries to understand potential intentions. The VirusTotal website, a collection of infected malware samples and anti-virus vendor labels, is one alternative. However, the security community has observed that anti-virus vendors each have their own malware naming scheme, leading to inconsistent labels [4, 5].

Techniques for online detection, such as function call analysis, can be another resource that reveals malware behavior when malware is executing. Taking the Windows API invocation calls as an instance, they provide access to system resources. Previous work [6, 7] shows that malicious behavior executed by malware involves one or more code sequences. A code sequence can correspond to one or more intentions; for example, plausible file creation and writing may be inferred to self-replicating. To analyze malware behavior and capture malicious actions, we focus on Windows malware and hook Windows API functions at the virtualization layer to intercept targeted malware activities at runtime and record the API calls that it invokes.

Over the last few years, there has been a dramatic increase in the number of publications on malware analysis using deep learning techniques. Algorithms such as deep neural networks (DNNs) [8, 9], convolutional neural networks (CNNs) [10, 11], and recurrent neural networks (RNNs) [10, 12] have been investigated for malware analysis, and show that input features obtained from statistical methods can be used to detect or classify unknown samples, and yield acceptable performance. Most applications consider only system operations such as read, write, or edit via system calls or API calls as input features. Parameters in an API call include important resource information, such as making connections via IRC. However, few studies have reported the effects of taking such parameters into consideration.

**Fig 1:**
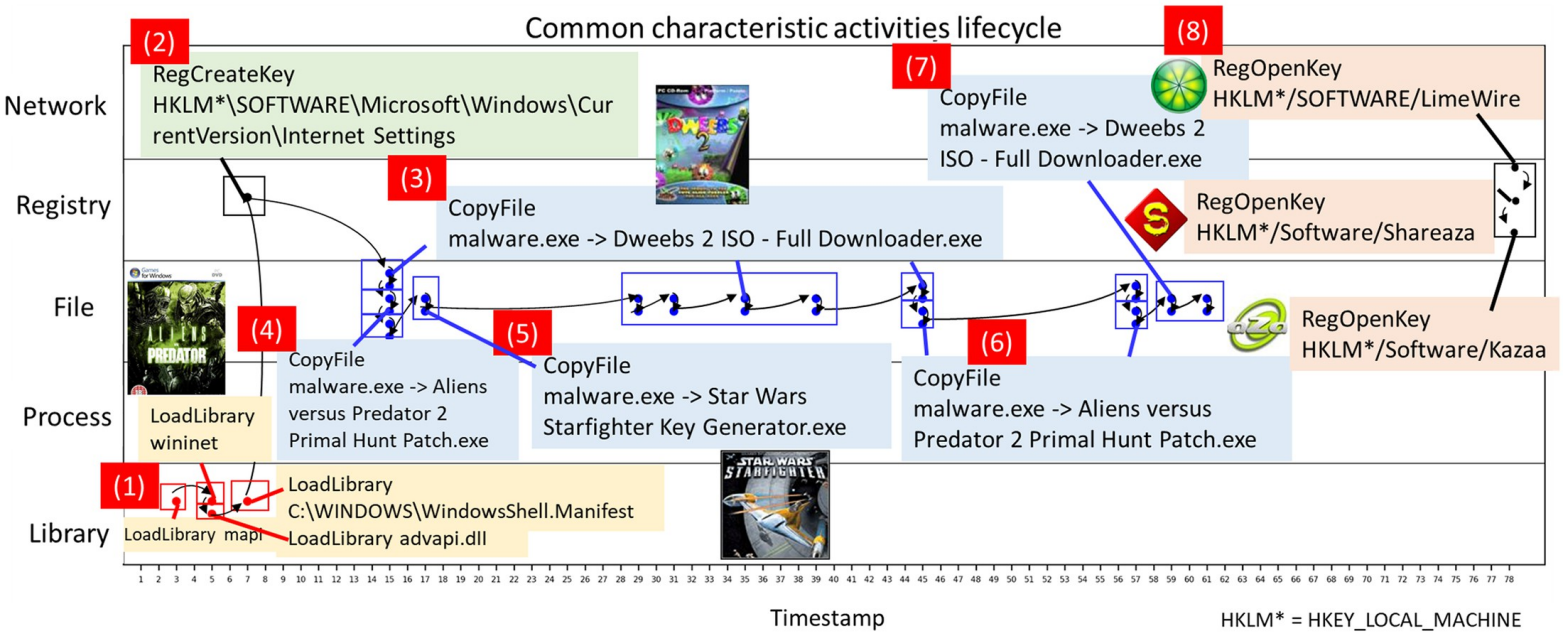
Sample life cycle of Eggnog malware.

To capture the essentials of the execution behavior of malware and to identify and characterize the intents during the course of the execution of the malware, we analyze a number of malware variants classified as the Eggnog family. Eggnog is designed to drop a large amount of portable and executable (PE) files into the folder “My Downloads” of the root folder by copying itself and attempt to spread itself via p2p software. The characteristic activities in the life cycle of this family are shown in Fig 1. For this family, all variants go through four stages (*LoadLibrary*, *RegCreateKey*, *CopyFile*, and *RegOpenKey*) during the execution lifetime. The behaviors from our observation are “trojan”, “dropper”, “PE”, “p2p”, “worm”, and “riskware” from (1, 3–7), (3–7), (3–7), (8), (8), and (1–8) in Fig 1 respectively. This illustrates three key observations. Firstly, accesses to system resources can reveal malware behavior. That is, system functions in (1), registry key for a browser security zone and privacy setting, a plausible file name with an EXE extension in (3-7), and peer-to-peer software in (8), deliver some meaningful messages related to malware intentions. Secondly, the information from the name of malware family is limited. It is difficult to grasp the characteristics of malware directly. Thirdly, a single program can perform a large number of API calls per execution. It is difficult to recognize malware behavior by examining each API call one-by-one. For example, “dropper” involves a series of operation of file access.

To overcome these challenges, we propose an attention-based neural sequence-to-sequence (seq2seq) model, called TagSeq, which examines Windows API call sequences and generates tags to describe malicious behavior. We take as input features not only API calls but also parameters (resources). Thus, to preserve the semantics of parameters, we propose three embedding modules to transform the Windows API function parameters, the registry, filenames, and URLs into low-dimension spaces. Also, TagSeq is developed with an attention mechanism, so that these tags can be used to explain malicious behavior via associated API call sequences. Our results show that TagSeq identifies the most likely malicious characteristics of a given malware program. This can help security administrators analyze malware more efficiently from the generated explainable descriptions.

Our major contributions include the following:

- First, we develop a neural network model which automatically predicts a set of tags to label malware behavior. The output generated helps security administrators analyze malware more efficiently, because the generated tags constitute a straightforward and easy-to-understand description of what the program does.
- Second, we propose methods to transform a Windows API call consisting of a function name, parameters, and a return value into a numerical representation. Our results demonstrate that TagSeq preserves closeness properties even for unseen API calls.
- Third, we show that attention maps obtained using TagSeq explain key characteristics from trace logs of given malware. This outcome helps security administrators to characterize the crucial behavior of given malware.
- Finally, we present a data collection procedure for pairs of Windows API calls and semantic descriptive tags. These collected tags yield a better understanding of malicious behavior in malware analysis.

## 2 Related work

### 2.1 Malware analysis

A large amount of literature exists on malware analysis [1–3]. Generally, this can be classified into static analysis [9, 13–19] and dynamic analysis [6–8, 10, 20–23]. Research on static analysis investigates malware behavior from binaries or source code, whereas studies on dynamic analysis examine execution activities after a device has been infected. Static analysis collects information from binaries or source code by decompressing or unpacking them, rather than executing them. Tesauro et al. [13] and SSornil and Liangboonprakong [18] use n-gram analysis to examine malware files as a sequence of hexadecimal values. Ravi and Manoharan [16] and Veeramani and Rai [17] study import APIs of PE files and analyze the occurrence frequency of each unique API call. Studies on dynamic analysis collect system calls or API calls to analyze malware behavior. Forrest et al. [20] collect system call sequences invoked by a program and use short sequences of system calls to represent normal program behavior to distinguish it from malicious behavior. Lee and Stolfo [21] utilize data mining methods to cluster malicious and normal system call sequences to distinguish between attacks and normal programs. Bayer et al. [23, 24] record Windows native system calls and Windows API functions, and propose a clustering algorithm to detect malicious behavior of the same type.

Recent years have seen a growing indication of the effectiveness of neural network techniques for malware detection and malware analysis. As an example of static analysis, Saxe and Berlin [9] incorporate features extracted from the binaries—contextual bytes, PE imports, string 2D histograms, and PE metadata features—into a deep neural network model, yielding low false-positive rates and high scalability. For dynamic analysis, Dahl et al. [22] consider trigrams of system API calls, combinations of a single API call and an input parameter, and patterns observed in process memory; reduce the input space by using random projections; and train a neural network model to identify files as malicious or benign. Huang and Stokes [8] combine multi-task learning with deep learning for binary classification (as malicious or benign) and malware family classification. Athiwaratkun and Stokes [25] treat API call events as characters, applying CNNs to learn character-based events and RNNs to learn hidden relations between events for malware classification. Čeponis et al. [26, 27] investigate the number of system calls with the different design of neural networks, such as LSTM, GRU, CNN, CNN-LSTM and CNN-GRU. Damaševičius et al., [28] consider either DNN or CNN layers to select representative features for the later malware detection. Most current studies concentrate on malware classification or detection problem or malicious patterns identification, as to what exactly the malware does, it still relies heavily on manual analysis for high-level semantic descriptions.

### 2.2 Malware behavior analysis

Many recent work consider malicious behavior recognition to distinguish malware from benign through the observation of execution traces. Sebastio et al. [6] construct behavioral heuristics and mined the common system call dependency graphs (SCDGs) as behavioral representation. Amer and Zelinka [7] compute the transition probability within Windows API call function names in both malware and benign. Both of their finding show that the extracted behavioral representation, either the resulting SCDGs or the transition sequences, can be seen as the characteristics of malware. The resulting behavioral representation of malware is proven to be significantly different from that of benign. However, these approaches only focus on the representative behavior identification but lack any semantic description for it. In this paper, we focus on associating easy-to-understand tags with malware behavior. The most similar studies for the same purpose are [29, 30]. Qiu et al. [29] consider API calls extracted from the raw binary files and metadata information of an app to infer malicious capabilities, and Huang et al. [30] analyze the parameters of API calls to generate malware annotations. TagSeq is different from [29, 30], as the generated description is tied to call subsequences.

### 2.3 Malware characteristic labeling

Except for the observation from malware static and dynamic analysis, raw labels from a number of anti-virus engines can provide their point of view of the characteristic of the target malware. This is because anti-virus vendors usually develop their own intelligent but closed naming convention, they seldom follow a standard naming convention, such as CARO [31] and CME [32]. Sebastián et al. [4], Hurier et al. [5], Zhang et al. [19] analyze the association among labels given from different anti-virus vendors to derive a unified label. Based on the inconsistency labels from anti-virus scanning reports, Sebastián et al. [4] design a heuristic algorithm to filter out generic tokens in a raw label and then to find alias name to link different labels; Hurier et al. [5] design a clustering algorithm to compute the common relatedness in pairs of an anti-virus engine and a family label, and then grouped similar malware family; Zhang et al. [19] treat labels as one of input features, and included it with static code analysis and meta-information of an app, to learn a malware representation for malware classification. These studies have shown the success of using massive labels from anti-virus scanning reports on malware labeling. Thus, in this work, we consider the use of the shared intelligence from anti-virus vendors, to label the characteristic of malware.

## 3 Malware execution profile and tags

**Fig 2:**
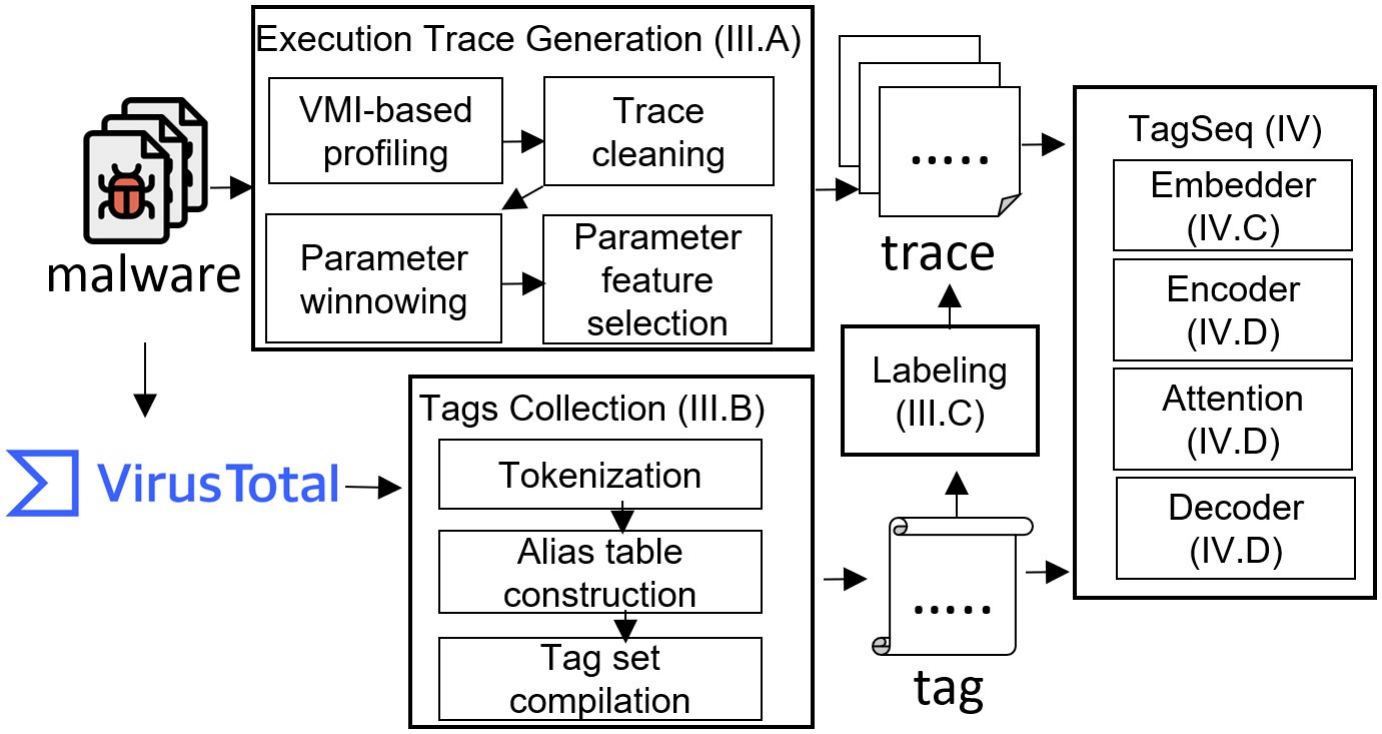
The workflow of TagSeq process.

When given a malware sample, the goal of TagSeq is to output a list of tags which capture the characteristics of the series of malicious activities. A high-level overview of the workflow is shown in Fig 2, including execution trace generation, tag collection, labeling and TagSeq neural network. Malware behaviors, i.e., API call sequences, are recorded by the execution trace generation, and the descriptions of the behaviors, i.e. tags, are gathered in the tags collection. Traces and tags are paired in the labeling stage. When given pairs of traces and tags, the TagSeq neural network is trained to label descriptive tags for unknown malware.

### 3.1 Execution trace generation

#### 3.1.1 VMI-based profiling

We use a dynamic malware behavior profiling system [33] to record malware execution traces. In the dynamic malware profiling system, 62 Windows API calls in five categories—library use, process invocation, file I/O, registry access, and network access—are hooked (shown partly in Table 1). In contrast to conventional dynamic behavior analysis systems in which only the API function name is recorded for profiling, in the system we also record parameters and return values. This helps users to understand, for example, what file is accessed or what process is opened. Moreover, since in Windows, configuration information for the operating system, services, applications, and user settings is stored in registries, registry-related operations are important in malicious behavior analysis; we are particularly interested in the parameter values. For instance, *RegQueryValue* is called by many programs to inspect system/application settings. The profiling system records the function name and its corresponding parameter values as well as the return value. Also, a malware sample may create or fork one or more processes. One execution trace is generated per process.

**Table 1:** Windows API function names and parameter types used in this study.

Category	API function name	Parameter name
Registry	RegCreateKey, RegDeleteKey, RegDeleteValue, RegOpenCurrentUser^+^, RegEnumValue, RegQueryValue, RegSetValue	hKey, lpSubKey, lpValueName
Process	CreateProcess, CreateRemoteThread, CreateThread, TerminateProcess, ExitProcess*, OpenProcess^+^, WinExec^+^	lpApplicationName, dwCreationFlags, uExitCode
Network	InternetOpen^+^, WinHttpConnect, InternetConnect, WinHttpOpen^+^, WinHttpOpenRequest^+^, WinHttpReadData^+^, WinHttpSendRequest^+^, WinHttpWriteData^+^, GetUrlCacheEntryInfo^+^, HttpSendRequest^+^	lpszServerName, pswzServerName, nServerPort
Library	LoadLibrary	lpFileName
File	CopyFile, CreateFile, DeleteFile	lpFileName, lpExistingFileName, lpNewFileName, dwCreationDisposition, dwDesiredAccess, dwShareMode

* Only “ExitProcess” has no return value.

^+^ Associated parameter values are not considered.

#### 3.1.2 Trace cleaning

The purpose of this work is to understand the important behavioral characteristics of malware from the recorded traces. We recorded Windows API calls for the first five minutes, yielding a large set of information. Shown in Table 1 are 28 selected API function names, their associated parameter types, and the return values. In addition, some malware makes the same API call repeatedly. Since the goal is to recognize distinct malicious behavior, only the first API call is retained in our trace.

#### 3.1.3 Parameter winnowing

**Fig 3:**
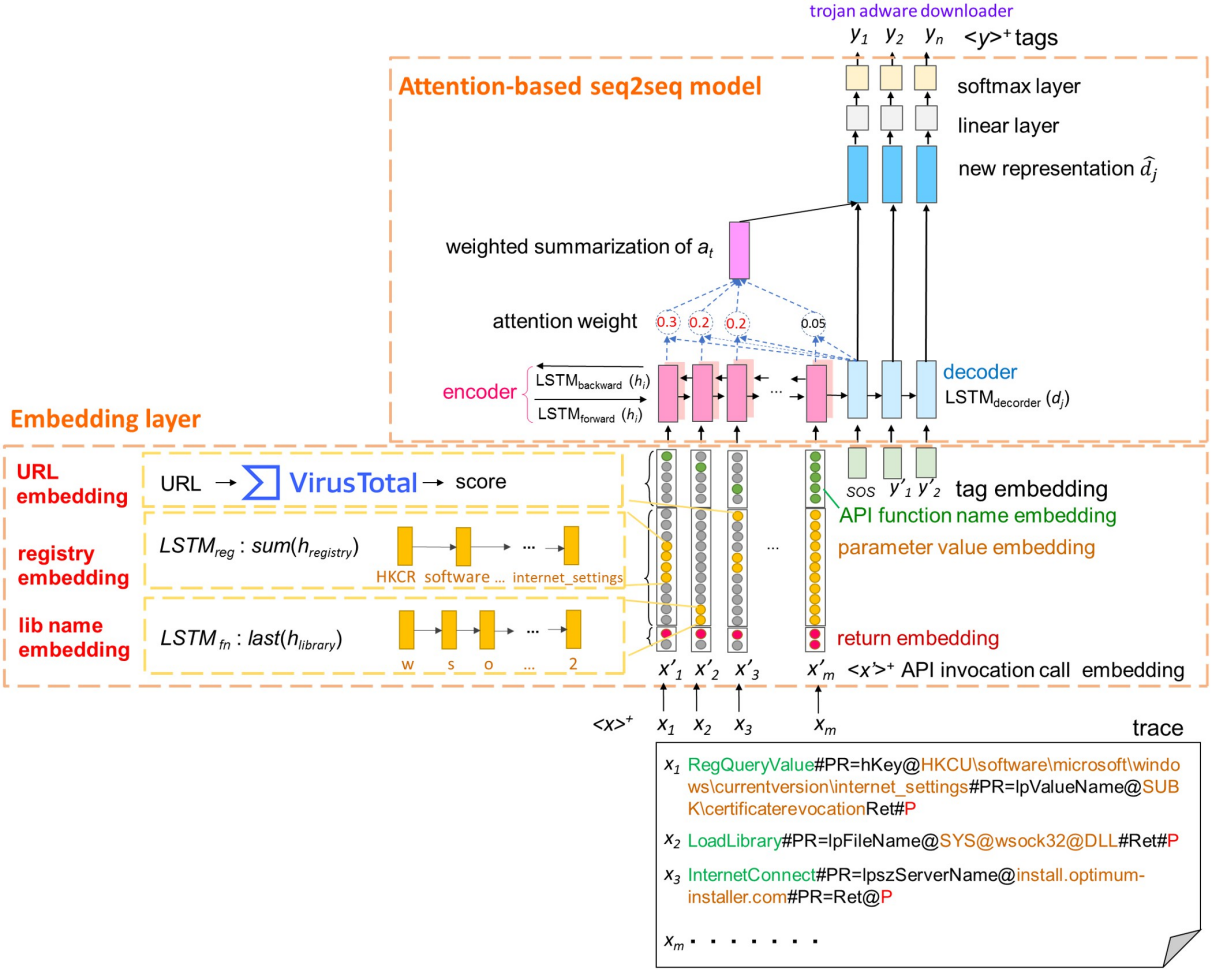
Given a profile which contains API calls *x*_1_…*x*_*m*_, the proposed system transforms a profile *x* = {*x*_1_…*x*_*m*_} into embedding vectors $x_{1}^{\prime },\ldots ,x_{m}^{\prime }$, and predicts a list of tags *y* = {*y*_1_…*y*_*n*_} by capturing the relations between each tag *y*_*j*_ and input sequence *x* = {*x*_1_…*x*_*m*_}.

Distinct malware with the same intent can have slightly different parameter values, such as user-profile folders “User’s Desktop” and “User’s Documents”, depending on the version of the operating system or the type of executable. To reduce such noise, file directory and registry key values are symbolized as described in [6]. Also, traces are reformatted and presented as line-by-line Windows API calls, as illustrated in the profile in Fig 3.

#### 3.1.4 Parameter feature selection

After winnowing, directory-related parameter types *lpApplicationName*, *lpExistingFileName*, *lpFileName*, and *lpNewFileName* are split into the parameter-directory and parameter-extension features. Only the library names of parameter type *lpFileName* from *LoadLibrary* are preserved.

### 3.2 Tags

We seek to collect a bunch of tags which are descriptive terms or keywords to help users quickly grasp the characteristics of a malware program. We consulted the labels from a number of anti-virus vendors on the VirusTotal website. Currently, these vendors label malware based on what they have found and what they seek to highlight. It is widely recognized that their labeling criteria are inconsistent and in many cases confusing. For example, a sample with SHA-256 value of 000e99 is labeled as WORM/Vobfus.CF, Gen:Variant.Chinky, and Win32/AutoRun.VB.AGQ by Avira, BitDefender, and ESET-NOD32, respectively. Nonetheless, these labels do reveal useful and interesting information about what a malware sample does. We implemented a program to collect the labels of malware samples, and took the following steps to extract a set of tags.

#### 3.2.1 Step 1: Tokenization

Labels were changed to lowercase and tokenized by delimiters “, ! () [] @: / . _ -”. Only the first and second tokens were considered. For example, “Gen:Variant.Kzay.73782” was transformed into “gen” and “variant.”

#### 3.2.2 Step 2: Alias table construction

We manually examined the set of tokens produced in Step 1 and built a table of alias names for the tag candidates with the same meaning. For example, tokens like “troja”, “troj”, “trj” and “tr” were considered abbreviations of “Trojan,” and “pua (potentially unwanted application)” is an alias of “pup (potentially unwanted program).”

#### 3.2.3 Step 3: Tag set compilation

After Steps 1 and 2, seventy-six tokens were finally compiled to form the set of tags shown in Table 2 for use in our automated malware tagging system.

**Table 2:** The collected seventy-six tags.

Categories	Tags
Type or large family	adware, backdoor, bot, browsermodifier, bundler, ddos, game, grayware, networm, PUP*, ransom, riskware, rootkit, spyware, trojan, virus, worm,
Behavior	autorun, binder, browserhijacker, clicker, crypt, dialer, dns, downloader, dropper, fakealert, fakeav, filecryptor, fileinfetor, flooder, fraudtool, hacktool, infostealer, installer, joke, keylog, lockscreen, memscan, monitor, packed, prochollow, procinject, virtool, webfilter
Route of infection	air, email, im, p2p, patch, pdf, proxy, sms, uds
Programming language	autoit, bat, html, js, php, vb
Other	android, apt, avt, constructor, exploit, FAT*, fca, hllp, iframe, irc, keygen, MBR*, MSIL*, password, PE*, rat

* These tags are changed to uppercase to make them easier to understand.

### 3.3 Labeling

Given this list of tags, we can label the trace files in the collected dataset. Note that in our analysis of the labels collected from VirusTotal, we observed that some labels contain malware family names. For these, we manually looked up their technical descriptions on the Internet and built a relationship table of family names and tags. For instance, a malware family “atraps” is a type of “trojan” which gathers confidential information from computers and sends it to a predetermined location. In the table, “atraps” is associated with tags “trojan” and “infostealer.”

For each trace file in the dataset, if any tokens in the set of the labels of the corresponding malware sample match anything in the tag set, the alias table, or the relationship table, they are collectively used to label the file. For example, “tr”, “psw”, “lbank”, and “f” are tokens from the label “TR/PSW.lbank.F”. Among these, “tr” and “psw” are respective aliases of “trojan” and “password”. Thus, the file is labeled “trojan” and “password”.

## 4 System design

We construct TagSeq which takes the execution trace of a malware sample as input and automatically produces as output a list of tags which describe the characteristic operations or intentions of the program.

### 4.1 Problem definition

We model our research problem that maps a series of API calls *x* = {*x*_1_, …, *x_m_*} to a list of tags *y* = {*y*_1_, …, *y_n_*}. The generated tags are represented as the potential characteristics of a given malware. Namely, the output is a list of tags which are selected from the tag set proper to reflect operations in the input trace. This is similar to answering yes-or-no questions such as “Is this ‘trojan’?”, “Is this ‘password’?”, etc. The conditional probability *p*(*y*|*x*) is decomposed as:

$$\begin{array}{c} p\left(y|x\right)=\prod_{t=1}^{n}p(y_{t}|y_{<t},x) \end{array}$$ (1)

where *y*_<*t*_ = {*y*_1_ … *y*_*t*−1_}.

Fig 3 depicts the TagSeq neural network architecture, which composed of an embedding layer and an attention-based sequence-to-sequence (seq2seq) model. The embedding layer processes the information on each Windows API call. Since the execution traces are text files, the plaintext representations of the API calls are transformed into vectorized representations in the embedding layer. An API call consists of a function name, one or more parameter values, and no or one return value. Thus, the corresponding embedding layer consisting of an API function name embedding, a parameter value embedding, and a return value embedding takes as input a variable-length execution trace *x* = {*x*_1_, …, *x_m_*} and outputs a sequence of embedding vectors $x^{\prime }=\left\{x_{1}^{\prime },\ldots ,x_{m}^{\prime }\right\}$. Note that in the parameter value embedding, there are three proposed embedding modules: registry value embedding, library name embedding, and URL embedding. Below we explain the three embedding modules in detail.

A sequence-to-sequence (also termed encoder-decoder) model is a neural network architecture which consists of an encoder and a decoder. This framework has been shown to be very effective and used in many recent advanced applications, such machine translation [34, 35], speech recognition [36], image segmentation [37, 38]. These tasks rely on a sequence of potentially varying lengths, and produces a sequential output. In our work, the encoder processes each API call embedding in a trace and outputs a sequence of vectors considering call dependencies. More specifically, the encoder processes a sequence of variable-length embedding vectors $x^{\prime }=\left\{x_{1}^{\prime },\ldots ,x_{m}^{\prime }\right\}$ and outputs a series of vector representations *h* = {*h*_1_, …, *h*_*m*_}. The decoder is conditioned on the output from the encoder, and learns to produce tags *y* = {*y*_1_, …, *y*_*n*_}. Since a generated tag associated with API call sequences from the encoder are desired, an attention mechanism is introduced to measure the relative importance of the input sequence and a generated tag. It computes the relation at between a tag and each hidden state *h*_*i*_ from the encoder to generate a variable-length sequence *y*.

### 4.2 Background

We use long short-term memory (LSTM) [39] units widely in TagSeq. An LSTM is a kind of recurrent neural networks (RNNs), designed to deal with sequential data. The main reason that we considered LSTMs is because it is designed to deal with variable-length and sequential data. That is, it can process varying length sequences and preserve information over many timesteps. More importantly, LSTMs can capture long-distance information and handle the vanishing gradients problem. LSTMs are used in TagSeq, including registry embedding, library name embedding, encoder and decoder. We use LSTMs to embed a registry is due to the hierarchical registry structure and a variable-length of a registry. The main reason of applying LSTMs for embedding library filename is to make a distinguish from the normal and malicious library filenames when the appearance of their filenames looks like similar. For the encoder, API calls are chronologically listed in the execution trace and the number of API calls varies dramatically, thus, applying LSTMs is a good choice. The purpose of using LSTMs for the decoder is to generate a list of variable-size tags without any pre-defined size. More details are presented as follows.

### 4.3 Embedding layer

An API Call *x*_*i*_ composed of API function name *w*_*i*_, one or more parameter values *v*_*i*_, and no or one return value *ret*_*i*_. The goal of the embedding layer is to produce a fixed-size vector as the corresponding embedding *x*′ when given a Windows API call *x*. Each element *x*_*i*_ is transformed to an embedding $x_{i}^{\prime }$ which is a concatenation of function name embedding $w_{i}^{\prime }$, parameter feature embeddings $v_{i}^{\prime }$, and return embedding ${ret}_{i}^{\prime }$. The closeness property is defined if the parameters are close in the original domain, and their embeddings are close in the embedding domain.

Here, each element learns its identical weighted matrix *E* (termed the embedding matrix).

$$\begin{array}{c} {x^{\prime }}_{i}=\text{concat}\;(E_{w}w_{i},\;{{\text{concat}}_{k}^{\left|pr\right|}\;(E}_{k}v_{ik}),\;E_{ret}ret_{i}) \end{array}$$ (2)

where *E*_*w*_ ∈ *R*^*e*_*w*_×|*w*|^, *E*_*k*_ ∈ *R*^*e*_*k*_×|*k*|^, and *E*_*ret*_ ∈ *R*^*e*_*ret*_×|*ret*|^ are the function name, parameter, and return embedding matrices respectively, and *e*_*w*_, *e*_*k*_, and *e*_*ret*_ are the respective embedding sizes.

An API call utilizes parameters to provide developers with access to the resources of a Windows system. Each type of resource can have a number of values with different properties, e.g., registry name and path, file name and path, and library name. For such large categorical values, it is computationally inefficient to model them all using standard one-hot encoding. We focus on three important types of resources—registry, filename, and URL—and propose respective approaches to transform an API call into a low-dimension vector while preserving semantics. The remaining input values, including the API function name, other parameter values, and the return value, are initialized by drawing samples from a uniform distribution with Xavier initialization [40], and then updated by backpropagation. Thus, their associated embedding matrices are constructed.

#### 4.3.1 Registry value embedding

The Windows registry is a hierarchical database which includes keys, subkeys, and values. A key is a node of the hierarchical structure, a subkey is a descendant node of a key, and a value is a name-data pair stored within a key. Keys may contain values and subkeys. When given a Windows registry parameter value, the registry embedding layer transforms it into a fixed vector as the registry embedding denoted by $v_{reg}^{\prime }$.

The structure of registry keys is similar to that of folders in the file system; thus they are referenced with a syntax similar to Window path names, using backslashes to indicate levels of hierarchy. Thus, we construct a registry value embedding module that tokenizes keys using the backslash, ‘∖’, and then use a LSTM unit referred to as LSTM_reg_ to transform a key denoted by *key* = {*key*_1_, …, *key*_*n*_} into hidden vectors $h=\{h_{key_{1}},\ldots ,h_{key_{n}}\}$. All hidden vectors are then summed to a registry representation $v_{reg}^{\prime }$. For example, a key “HKCR∖software∖microsoft∖windows∖currentversion∖internet _settings” contains six tokens: “HKCR”, “software”, “microsoft”, “windows”, “currentversion”, and “internet_settings.” Each token is an input to the LSTM unit. The output hidden vectors constitute the registry key representation, i.e., *h*_HKCR∖software∖…∖internet_settings_ = *h*_HKCR_ + *h*_software_ + … + *h*_internet_settings_. The intuition behind this equation is that each token can make contribution to the final representation of a registry. In this way, we preserve the hierarchical relation between tokens and ensure a fixed and consistent embedding size regardless of the number of keys.

#### 4.3.2 Filename embedding

From our analysis of malware operations, malware programs often code filenames with spellings deformed from familiar names to obfuscate their purposes, e.g., “2dvaai32.dll” (LoadLibrary#PR=lpFileName@SYS@2dvaai32@DLL#PR=Ret@N) and “3dvabi32.dll” (LoadLibrary#PR=lpFileName@SYS@3dvaai32@DLL#PR=Ret@N) vs. “advapi32.dll”. Similarly, there are filenames which comprise a filename and a random number, e.g., “tsu08c6ec63.dll” (LoadLibrary#PR=lpFileName@USR@tsu08c6ec63@DLL#PR=Ret@P) and “tsu0ac63fe4.dll” (LoadLibrary#PR=lpFileName@USR@tsu0ac63fe4@DLL#PR=Ret@P). Some filenames are generated from hash values, e.g. “518ca2bf37e13.dll” (LoadLibrary#PR=lpFileName@USR@518ca2bf37e13@DLL#PR=Ret). Thus, the possible combinations for filenames are numerous and unpredictable.

Given a filename as the parameter value, the filename embedding layer transforms the name into a fixed-size vector as its embedding, denoted by $v_{lib}^{\prime }$. Here, we separate the filename into a sequence of character strings {*c*_1_, …, *c_n_*} and input each character string to a LSTM_fn_ unit one by one to obtain the corresponding hidden vectors $\left\{h_{c_{1}},\ldots ,h_{c_{n}}\right\}$. The last hidden state $h_{c_{n}}$ is taken as the filename representation $v_{lib}^{\prime }$ (The sum of each hidden vector was also considered when we implemented the system, but the last hidden vector works better). For example, filename “wsock32” can be split into the series of characters, {*w*, *s*, *o*, …, 2}. Each letter is an input to the LSTM_fn_ unit. They are transformed into the associated hidden vectors, i.e., *h*_*wsock*32_ = {*h*_*w*_, *h*_*s*_, …, *h*_2_}, where *h*_2_ can be considered the filename representation for ‘wsock32’. The merit of the proposed LSTM unit is that it captures similarities between purposely obfuscated file names or different variations of the same filename while treating each individually.

#### 4.3.3 URL embedding

Malware programs often include code to visit remote malicious web sites in the background and gain control of a host without being detected. However, it is difficult to distinguish from the bare text of an URL whether it is malicious. Nonetheless, we consider URLs to constitute important information about the program’s operations. Specifically, we consider URL reports from VirusTotal, which include the ratio of antivirus engines that detect a scanned URL as being malicious. This ratio is used as the score for the URL embedding. For example, the URL “install.optimum-installer.com” yields a ratio of 6:66. Since the score is a real number, the associated embedding E_URL_ is an identity matrix of 1×1.

### 4.4 Attention-based sequence-to-sequence model

In the sequence-to-sequence model, LSTMs are used as the encoder and the decoder. An encoder is used to process each API call embedding, and a decoder is employed to generate a variable-length list of tags. An attention mechanism is also applied to capture the relations between tags and API call embeddings.

#### 4.4.1 Encoder

The encoder is bi-directional LSTMs, which consists of two independent LSTMs. One processes each API call embedding from the beginning of a given trace to the end of the trace, and the other from the end to the beginning. Combining the outputs of the forward and backward networks can capture both the left and right contexts of an API call at each timestamp. A LSTM_forward_ encoder processes one API call embedding at a time, and outputs a hidden state from the current observation $x_{t}^{\prime }$ and the previous state $\vec{h_{t-1}}$.

$$\begin{array}{c} \vec{h_{t}}=\vec{{\text{LSTM}}_{\text{forward}}({x^{\prime }}_{t},\;h_{t-1})} \end{array}$$ (3)

LSTM_forward_ preserves the order of the API calls in the trace. In other words, the LSTM hidden state $\vec{h_{t}}$ at time *t* is indeed the result of processing the API call embeddings from the first to the current API call embedding $x_{t}^{\prime }$, i.e., $x_{1}^{\prime },\ldots ,x_{t}^{\prime }$.

Since malicious activities typically depend on the surrounding context, the current event at time *t* could be contextually dependent on the previous observation at time *t* − 1 and the next observation at time *t* + 1. We utilize a backward chaining LSTM_backward_ in addition to the forward chaining LSTM_forward_ for another perspective on the information: from the end to the beginning.

$$\begin{array}{c} \overset{\leftarrow }{h_{t}}=\overset{\leftarrow }{{\text{LSTM}}_{\text{backward}}(x_{t}^{\prime },h_{t+1})} \end{array}$$ (4)

The resulting forward and backward hidden vectors are then concatenated as the summarization at time *t*. The idea behind this design is for the encoder to perform forward and backward chaining of API calls in the trace embedding.

$$\begin{array}{c} h_{t}=\text{concat}\left(\vec{h_{t}},\overset{\leftarrow }{h_{t}}\right) \end{array}$$ (5)

The single representation can serve as the basis of the trace. Compared to the single directional forward LSTM, which contains more information about the end of API call than its beginning, the bidirectional LSTM considers information from both sides and passes that as an input for the following processing instead. This leads to a better representation behind a sequence of API calls in a trace.

#### 4.4.2 Decoder

Once the encoder encodes all embedded API calls in the trace, a LSTM_decoder_ outputs a variable-length list of tags conditioned on the trace representation from the encoder. Note that the number of tags in the output of the LSTM_decoder_ depends on the contents. At each time step *t*, the LSTM_decoder_ observes the previous predicted tag embedding $y_{t-1}^{\prime }$ and the previous hidden state *d*_*t*−1_ and computes the hidden state *d*_*t*_ as

$$\begin{array}{c} d_{t}={\text{LSTM}}_{\text{decoder}}\left(y_{t-1}^{\prime },d_{t-1}\right) \end{array}$$ (6)

Here, $y_{t-1}^{\prime }$ is initialized with Xavier initialization [40], and learns its own embedding parameters; *d*_0_ is the last hidden state *h*_*m*_ from the encoder.

One key component of the task is to align API calls to each tag. For instance, some code subsequences directly reflect the self-propagation operation, i.e., the “worm” tag. We apply an attention mechanism to identify such relations. We seek to pay more “attention” to the relevant motif(s) as we label. The decoder at each time step focuses on a different part of the input trace to aggregate the semantic information to produce the proper tag. There are two benefits to be gained. First, attention mechanisms in a neural network model can learn alignments between two objects, such as speech frames and text in speech recognition [41], two languages in machine translation [35, 42], and an image and its corresponding caption in computer vision [43]. This has been also applied to malware analysis [11] to visualize important region of byte sequences. Thus, we adopt attention mechanism to align a generated tag to each API call. Second, the mechanism can measure the relevance between two objects. Considering the relevant information can make better prediction. Thus, an attention mechanism is used to reveal the insights which API calls contribute to the tag prediction and provide the semantic meaning associated with the API calls in our study. Many attention variants, such as Bahadanau’s additive attention function [41] and Luong’s multiplicative style function [35], have been developed to integrate encoder-side information into the decoder at each time step. Here, the attention distribution is calculated as in [39] (Bahdanau’s additive attention was also applied when we implemented the system, but Luong’s multiplicative attention slightly performed better).

$$\begin{array}{c} w_{hd}=\frac{\text{exp}\left(\text{score}(h,d)\right)}{\sum_{i=1}^{m}\text{exp}(\text{score}\left(h_{i},d\right)} \end{array}$$ (7)

$$\begin{array}{c} \text{score}\left(h,d\right)=h^{T}W_{c}d \end{array}$$ (8)

where *W*_*c*_ is a weight matrix and attention distribution *w*_*hd*_ is a probability distribution over the input Windows API calls. The distribution tells the decoder which API calls matter to produce the next prediction. Given these attention weights, we compute a weighted summarization of the hidden states from the encoder:

$$\begin{array}{c} a_{t}=\sum_{i=1}^{m}w_{hd}h_{i} \end{array}$$ (9)

Given the weighted summarization of the hidden vectors at from the encoder and the hidden vector *d*_*t*_ from the decoder, a new representation $\hat{d_{t}}$ is the concatenation of *a*_*t*_ and *d*_*t*_ to compute the probability distribution over tags:

$$\begin{array}{c} y_{t}=\text{softmax}\left(\text{linear}\left(\hat{d_{t}}\right)\right) \end{array}$$ (10)

Here, a linear layer projects the new presentation $\hat{d_{t}}$ into a prediction layer, and a softmax layer computes the tag distribution. The predicated tag is the target class with the highest probability.

### 4.5 Training

Our goal is to maximize the likelihood of the predicted tags given a series of API calls as input. That is, when a training set of trace-tag pairs *S* is given, the training objective is to minimize the negative log-likelihood of the training data with respect to all parameters:

$$\begin{array}{c} L=-\sum_{i=1}^{S}\text{log}\;p(y^{i}|x^{i};\theta )=\sum_{i=1}^{S}\sum_{j=1}^{|y^{j}|}\text{log}\;p(y_{j}^{i}|x^{i},y_{<j}^{i};\theta ) \end{array}$$ (11)

where *θ* is the set of the model parameters, each (*x*, *y*) pair is a (Windows API calls, tags) pair from the training set, and *p*(*y*|*x*) is calculated as shown in (1).

### 4.6 Inference

We predict each tag for an execution trace x by:

$$\begin{array}{c} \hat{y}={\text{argmax}}_{y}p\left(y|x\right) \end{array}$$ (12)

Algorithm 1 concludes the operations of TagSeq neural network model described above.

**Algorithm 1** TagSeq Neural Network

**Input**: an execution trace ***x***

**Output**: a set of tags ***y***

1: **while** *θ* not convergences **do**

2:  **Forward Propagation**:

3:  ***x***′ ← Get API call embedding in (2)

4:  ***h*** ← Get encoder hidden state in (5)

5:  ***d*** ← Get decoder hidden state in (6)

6:  ***w*** ← Compute attention weights in (7)

7:  ***a*** ← Compute attentive decoder in (9)

8:  ***y*** ← Generate tags in (10)

9:  **Backward Propagation**:

10:  conduct backward propagation in (11) with Adam;

11: **end while**

12: # Use the trained network to discover tags ***y*** in (12) of an execution trace ***x***

## 5 Evaluation

### 5.1 Dataset

We collected 11,939 malware samples (Except for malware samples, all data will be available in public when the paper is accepted.) from NCHC’s OWL project (https://owl.nchc.org.tw). In practice, we excluded some profiles contain too many (300) or too few (10) API calls. For the situation with less than 10 API call invocations, it is usually involving system environment setting problems, and for a high number of API invocations, the malware likely runs into some recurring events. As malware in both situations cannot provide useful information, we exclude them from evaluations. As shown in Table 3, the final dataset includes 14,677 profiles (9,666 samples).

**Table 3:** Sample and profile statics.

Categories	Samples	Profiles
Original	11939	19987
Uninterpretable encoding	3	3
No tag	91	140
Length >300 or Length <10	2179	5167
Final	9666	14677

Labels from VirusTotal were crawled in April 2018. Based on these labels, we labeled the tags for each malware sample. If a sample had any child process file, it was labeled with the same tags as the main process. We also sorted the tags in descending order by frequency to control the variance from the tag order, to ensure that frequent tags are predicted first. The frequency refers to the number of traces annotating the tag. We observe that high-frequent tags represent broad categories, such as malware types, which are output first to give a broad-to-narrow sense. We randomly split the dataset into a training set (80%), a development set (10%), and a testing set (10%). If the number of tags in the entire dataset was less than 10, it was distributed to the three sets based on a uniform distribution. Distributions of the three sets were then validated by F-test until none had significant differences. The description for the distributions of the three datasets is shown in Table 4. Results is reported on the testing set.

**Table 4:** Dataset statics.

	Train	Valid	Test	Total
Samples	7718	975	973	9666
Profiles	11684	1512	1481	14677
Average number of API calls	92.89	91.20	93.44	92.77
Standard deviation of API calls	70.55	71.47	72.03	70.79
Average number of tags	7.44	7.38	7.41	7.43
Standard deviation of tags	2.63	2.55	2.55	2.61
Maximum number of tags	17	15	15	17

### 5.2 Implementation details

Model hyper-parameters were selected on the validation set. Optimization was performed using the Adam optimizer [44] to update the parameters, with an initial learning rate of 0.0002. We ran the training for 600 epochs. We started halving the learning rate at epoch 300, and then decayed it every 100 epochs. We set the number of layers of LSTMs to 2 in both the encoder and the decoder, and we set each LSTM hidden unit size to 256. The mini-batch size for the update was set at 16, and the dropout probability for regularizing the model was set to 0.1.

### 5.3 Baselines and model variations

We compared the performance of TagSeq and other methods to answer two research questions: (1) Can the parameter embedding or the return embedding help models to predict tags? (2) What are the effects when applying different neural network models with the proposed embedding modules?

- Machine learning models: Five conventional machine learning methods include LinearSVC (Linear Support Vector Classifier), Random Forest, Decision Tree, GaussianNB (Gaussian Naive Bayes), and KNeighbors (K-nearest Neighbors) in Scikit-learn [45]. The machine learning based approaches are commonly used in malware analysis [46, 47]. As traditional machine learning methods generally do not accept a complete execution trace as input, we took the first five hundred API calls (with API categories and API function names only) of an execution trace and used PCA (principle component analysis) [48] to reduce the dimensions of the execution trace. The reduced API call sequences and associated tags were used as input.
- Convolutional Neural Network (CNN): Following the design of TagSeq, the model had the same embedding layer but replaced the proposed attention-based encoder with an attention-based convolutional neural network. Three convolution layers (256, 192, 64) with an average pooling layer were used. It connected to a dense layer and a sigmoid layer. More details are found in [40].
- TagSeq (LSTM + MLC): The task is a multi-label multi-class problem, mapping one sample to one or more tags. Following the design of TagSeq, the MLC model had the same embedding layer and encoder and outputed the final hidden state, the decoder, however, was replaced with a linear layer and a sigmoid layer.

For each model, three input variations—the API function names only, the names plus the associated return values, or the names plus the returns and the parameter features—were evaluated. To ensure that the performance was not simply due to an increase in the number of model parameters, for the convolutional neural network (CNN), TagSeq (LSTM + MLC) and TagSeq (Seq2Seq), we kept the total size of the embedding layer fixed at 256. Table 5 lists the embedding size used for Windows API calls. Please notice that only deep learning approaches included parameter embedding since they were learned based on the proposed TagSeq framework.

**Table 5:** Embedding sizes for three configurations.

ID	Input Variations	Name	Return	Parameter
I.	API function name	256	-	-
II.	API function name + return	254	2	-
III.	API function name + return + parameter	50	2	204

### 5.4 Evaluation metrics

Recall and precision are used for evaluation. Recall is the preferred evaluation metric, because a high ratio of correctly predicted tags to the ground truth means most malicious patterns are found, which is very helpful for security analysts. Precision is also reported to show the percentage of correctly predicted tags and the number of all predicted tags. Note that the predicted tags were counted as a group when a profile had child processes.

- Recall denotes the fraction of the predicted tags that are correctly estimated over the total amount of ground truth tags. It reflects how close the prediction is to the expert-annotated tags.
  $$\begin{array}{c} Recall=\frac{|\hat{y}\cap y|}{\left|y\right|} \end{array}$$ (13)
- Precision denotes the fraction of the estimated tags that are predicted correctly. It represents the ability of a classifier to identify malicious intent.
  $$\begin{array}{c} Precision=\frac{|\hat{y}\cap y|}{|\hat{y}|} \end{array}$$ (14)

### 5.5 Results

Table 6 presents the results of different models and input settings. In general, the performance of the deep learning-based methods are better than that of machine-learning based approaches among different input variations, especially TagSeq (LSTM + MLC) and TagSeq (Seq2Seq) have obvious impact. With respect to recall, the predictions from the TagSeq(seq2seq) model approximate the tags collected from the dataset. For precision, however, the percentage of correctly predicted tags from the TagSeq(LSTM + MLC) models is the highest, but the average number of predictions is far fewer than that of the ground truth (7.41). We compared the predicted tags from the TagSeq(LSTM + MLC) models to those from the TagSeq(seq2seq) models: 84% of the tags from the TagSeq(LSTM + MLC) models and 52% from the TagSeq(seq2seq) models are the same. For each model, the three input variants yield slightly different results. Generally, the performance in Table 6 suffers from a wide range of number of tags labeled by malware samples.

**Table 6:** Experimental results for different models.

Input Variation ID	I			II			III		
Model	P	R	|Tags|	P	R	|Tags|	P	R	|Tags|
GaussianNB	56.06	25.21	19.14	58.10	26.35	18.89	-	-	-
Decision Tree	40.34	55.13	5.81	41.96	53.06	6.20	-	-	-
Kneighbors	40.94	56.33	5.35	43.46	57.35	5.49	-	-	-
Random Forest	39.85	74.13	3.75	39.90	74.85	3.72	-	-	-
LinearSVC	41.26	68.86	4.29	41.80	70.01	4.28	-	-	-
CNN	42.72	69.32	4.47	40.35	72.27	4.06	40.82	69.63	4.30
TagSeq (LSTM + MLC)	45.50	72.39	4.46	44.66	74.10	4.34	46.40	72.10	4.63
TagSeq (Seq2Seq)	57.10	53.02	7.92	56.25	52.39	7.86	57.18	53.05	7.88

**Fig 4:**
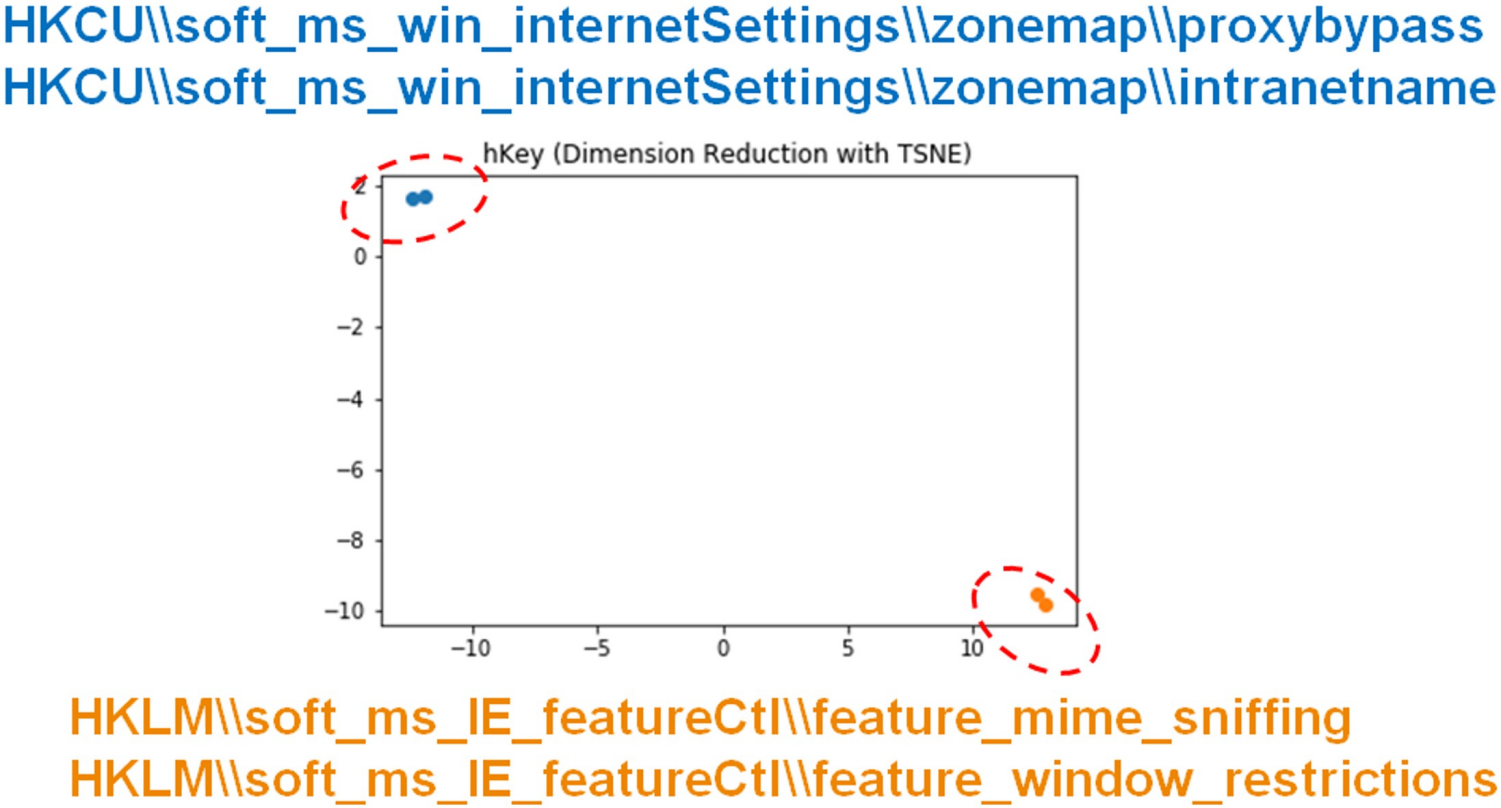
Registry embedding.

**Fig 5:**
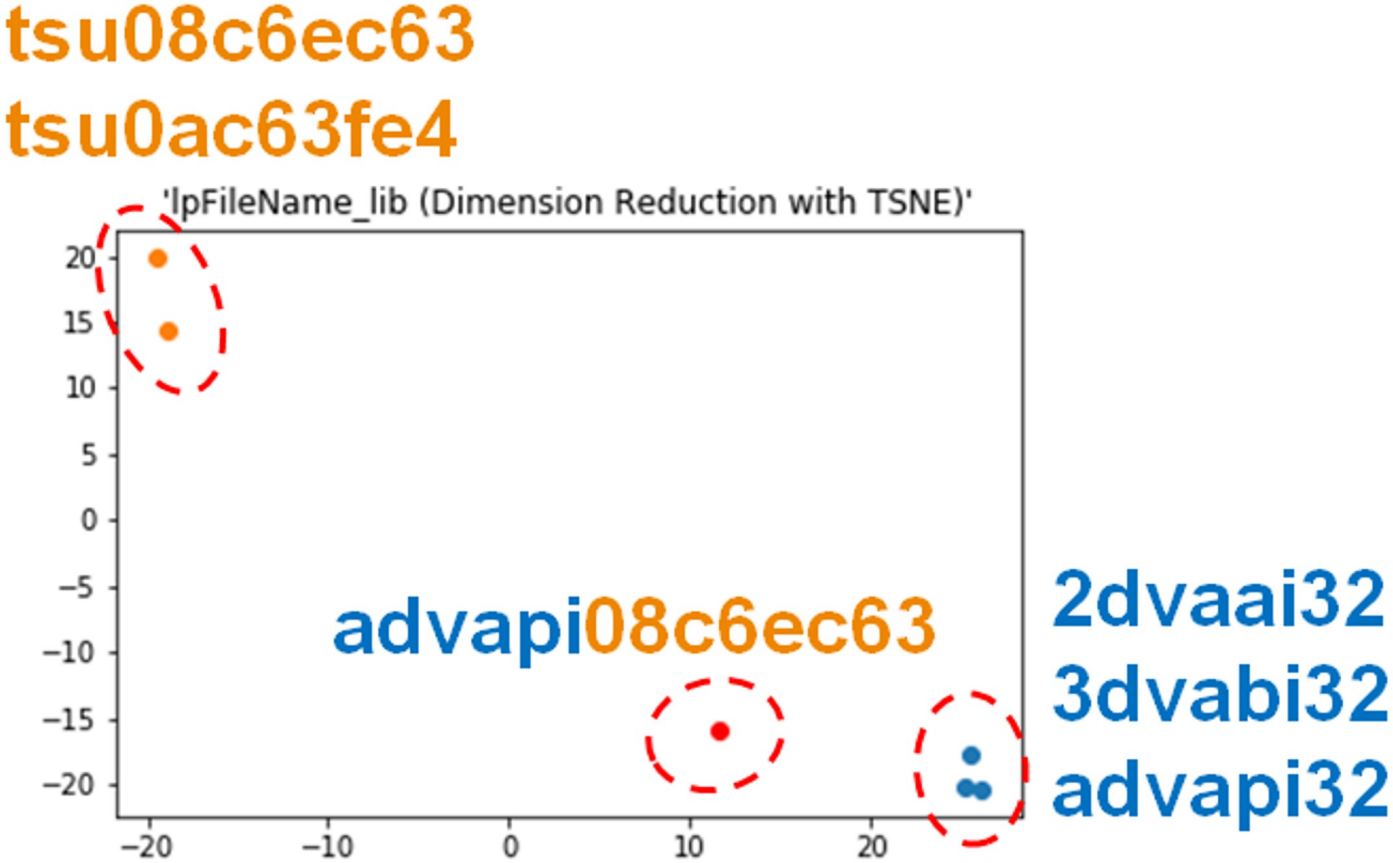
Filename embedding.

We demonstrate how the proposed embedding modules work in our proposed framework. Two examples from the registry value embedding module and the file name embedding module respectively are illustrated in Figs 4 and 5. The embedding values are transformed into 2-dimensional vectors using t-SNE [49]. In Figs 4 and 5, the parameter values outside of the box are selected from training set, and the parameter inside the box is from the testing set. Parameter values with the same sub-values, such as registry subkeys or characters, are located closely to each other, while distinct values are far from each other. Moreover, the registry value from the testing set, “HKLM∖soft_ms_IE_featureCtl∖zonemap∖ intranetname”, has two tokens that are the same as the parameter values in orange (HKLM∖soft_ms_IE_featureCtl∖*), and two other tokens that are the same as the parameter values in blue (HKCU∖soft_ms_win_internetSettings∖zonemap∖intranetname). Because the hierarchical relations are preserved, it is located close to parameter values with the same ancestor keys, instead of the same subkeys. Similarly, the filename value from the testing set, “advapi08c6ec63”, also maintains its physical relation in the space, that is, close to “advapi*” instead of the random number “tsu08c6ec63”. These results support our claim that the modules are able to process unseen data and preserve semantic structure in the numeric embedding space.

To summarize, considering both API function name and return values, or all three (API function name, parameter values, and return values) yields better performance than considering API function names only. The TagSeq model considering all three inputs captures the most likely malicious intentions. This achieves our main purpose, that is, the reduction of the workload of human analysts.

## 6 Comparison with existing system

Can the generated tags help for malware detection if they are seen as the characteristics of malware? To demonstrate the potential usefulness of the tags, we perform an extended malware detection based on the output tags from TagSeq. One existing system, AVClass [4], is used to make a comparison. It is an automatic malware labeling system based on the labels from a collection of anti-virus vendors in VirusTotal.

We additionally collected 440 benign execution files under the directory “%SystemDirectory%” for the malware detection experiment, and randomly divided both benign (440) and malware samples (9666) into training (80%), validation (10%), and testing (10%) set. We implemented our malware detection with different classification algorithms, including a neural network classifier, LSTM, and a traditional classification algorithm, SVM. When given the generated tags from TagSeq, the tags were represented as one-hot encoding and fed into the classification algorithms. Considering the highly imbalanced classes in our dataset, we fitted the models with different weights for each class to penalize mistakes on the minority class. The weights were based on an amount proportional to the number of samples in each class.

**Fig 6:**
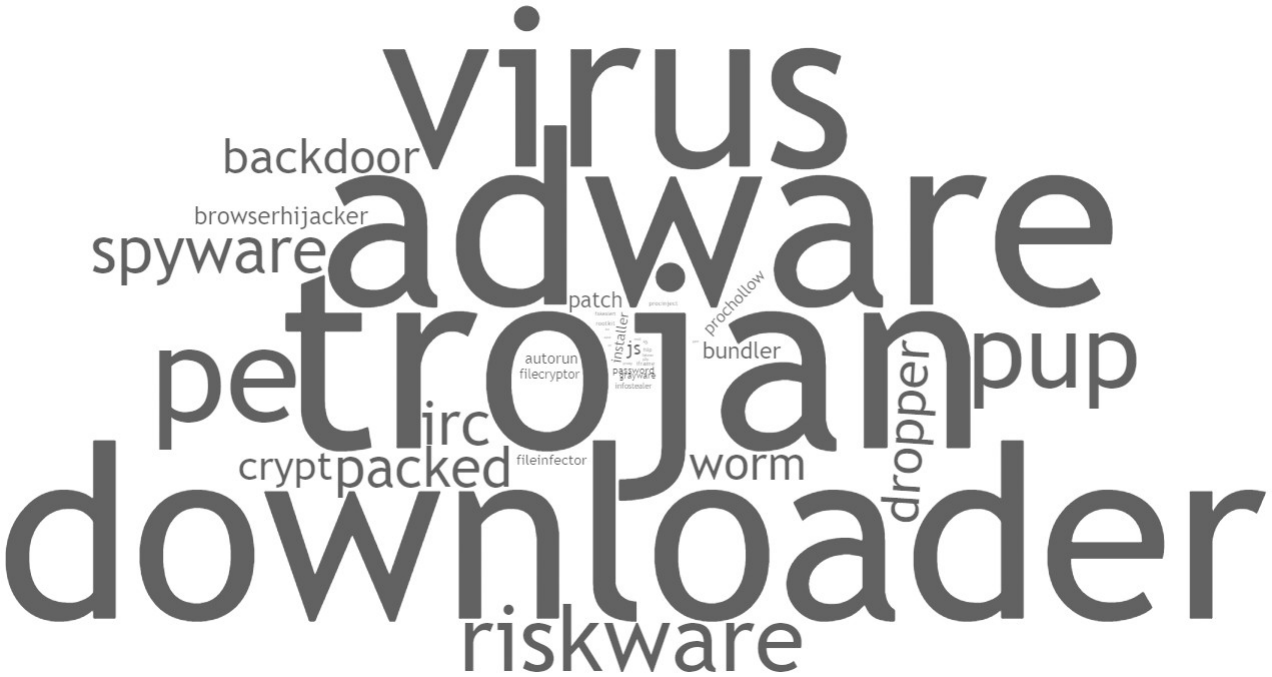
The tag cloud of ramnit.

Table 7 showed that models trained with the tags generated from TagSeq achieved 99% for recall and micro-F1, which were better than the results of AVClass, while their precision scores were slightly less than that of AVClass. One of the possible reasons was that all tags were designed for describing malicious behavior. Note that we do not claim that TagSeq is superior to the existing malware labeling system. Rather, TagSeq is designed to describe possible behavior of malware. Our goal of the comparison is to demonstrate that the generated tags can provide additional information for malware detection. More specifically, tags can be one of the features that fuse and unify the diverse labels from Anti-Virus engines. For instance, a tag cloud in Fig 6 is produced based on the generated tags from a family, ramnit. It holds the main characteristics like virus and trojan, and also able to infect files to open a backdoor to download or drop malicious PE files. Bigger word in Fig 6 represents greater weight. This shows that TagSeq can not only detect these samples in the family as malicious but also describe their properties with confidence.

**Table 7:** Comparison with different classifiers.

System	Recall	Precision	Micro-F1
AVClass	91.14	99.60	99.23
TagSeq+SVM	**99.63**	90.00	**99.27**
TagSeq+LSTM	**98.66**	93.84	**99.54**

## 7 Case study

**Fig 7:**
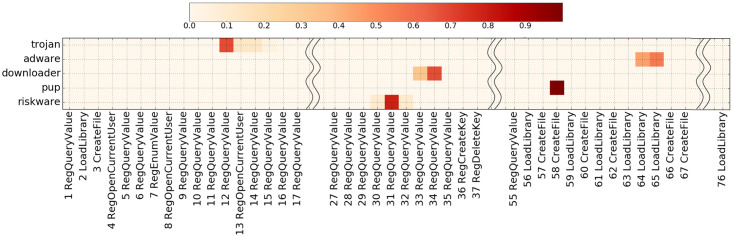
An attention map shows the predicted tags and associated motifs of a malware program (“Trinity”) after recording Windows API calls.

In this section, we use a case study to analyze the motifs, highlighted by the attention weights, to answer two questions: (1) Does a motif refer to malicious behavior? and (2) Can a tag explain a motif? We present behavior from a malware program called “Trinity” in VirusTotal. This is considered a potentially unwanted program (PUP) type of malware, which compromises privacy or weakens a computer’s security. Eleven labels were collected from anti-virus vendors: Win32:FourShared-C[PUP], TROJ_GEN.R47H1DP, Trojan.Downloader.gen, and Adware.Toolbar.111 by Avast, TrendMicro, VBA32, and DrWeb respectively. During the tag construction procedure, the malware was tagged as “trojan”, “adware”, “riskware”, “downloader”, and “pup.” Fig 7 shows the attention map of the optimal seq2seq model: each tag’s motif is clearly distributed in the profile. We explain these motifs in chronological order:

- Trojan: explores the user environment and system settings.
- Riskware: investigates system settings such as the path for the Windows service pack and the driver cache.
- Downloader: probes device paths, logs, and error reporting to observe whether its malicious behavior has been noticed.
- Pup: enables network access and named pipes, anonymously uses SMB or RPC protocols to invoke programs from other computers.
- Adware: loads a malicious execution file as a library file.

From our observation, the “trojan”, “riskware”, and “downloader” motifs involved investigating the system environment and occurred before the malware program executes malicious files. These could be taken as evidence showing these motifs could be a red flag, with a surrounding context of malicious behavior. As the actual malicious actions would be too random to be caught by the neural network model, it would pay attention only to initial API calls such as these. The “pup” and “adware” motifs are malicious actions. These findings support our expectation that a motif represents malicious intentions. It is possible that tags and motifs may frequently co-occur in profiles, which explains why the neural network catches the motifs with the tags. However, can tags describe the motifs? This need to be handled carefully, as the results might reflect the part of data that is collected. In this example, “pup” and “adware” represent multiple actions. Thus, we could assume that malware like pup and adware would include these actions, but not this action only.

## 8 Conclusion

In this paper, we present a novel neural TagSeq system to examine Windows API calls and produce tags to label the malicious behavior of malware programs. Results show that TagSeq taking an input with the API function names, the associated return values, and the corresponding parameter features can find the characteristics most likely to be malicious with respect to the number of tags and a high ratio of correctly predicted tags to the ground truth. This can help security analysts to understand potential malware behavior with easy-to-understand descriptions.

This study is a first exciting attempt to explore the behavior of malware using the proposed neural network model. Still, TagSeq has several limitations. Firstly, as a supervised learning-based method, TagSeq requires labeled data for learning the characteristics of malware. With sufficient pairs of malware and tags, the model can be robust and accurate. Secondly, TagSeq is designed to discover malware behavior based on the observed execution trace. Since some of the malware samples may have the ability to obfuscate, anti-debugging or anti-VM, it may affect the evaluation results of TagSeq. Investigating obfuscated malware activity is orthogonal to this work and an interesting future work. Another limitation is that the source of tags relies solely on VirusTotal website. The existing tags from VirusTotal only describe the malware instead of the detailed execution of malware. Such as function call-level tags can be required for better understanding malware behavior. With more reliable sources other than VirusTotal, accurately labeling from multiple sources is another challenge.

These findings suggest several dimensions that might profitably be addressed by future researchers in the field.

The first is the scalability. This results show that malware behaviors can derive from function calls and its parameters. Similarly, the core idea can be extended to other operating systems, in which a potential work can embed system calls to tag malicious behavior. The second is the input sources. The study presents that malicious behavior during execution tying to function calls can be observed from dynamic analysis. In similar fashion, some behavior may be also inferred from signatures based on static analysis. The third is the tag granularity. Malware intentions could involve many actions. When designing tags, it may be wise to use fine-grained tags for actions and coarse-grained tags for intent.

## References

[1] Egele M, Scholte T, Kirda E, Kruegel C. A survey on automated dynamic malware-analysis techniques and tools. ACM computing surveys (CSUR). 2008;44(2):1–42. doi: 10.1145/2089125.2089126

[2] Gandotra E, Bansal D, Sofat S. Malware analysis and classification: A survey. Journal of Information Security. 2014;2014.

[3] Ucci D, Aniello L, Baldoni R. Survey of machine learning techniques for malware analysis. Computers & Security. 2019;81:123–147. doi: 10.1016/j.cose.2018.11.001

[4] Sebastián M, Rivera R, Kotzias P, Caballero J. Avclass: A tool for massive malware labeling. In: International symposium on research in attacks, intrusions, and defenses. Springer; 2016. p. 230–253.

[5] Hurier M, Suarez-Tangil G, Dash SK, Bissyandé TF, Le Traon Y, Klein J, et al. Euphony: Harmonious unification of cacophonous anti-virus vendor labels for android malware. In: 2017 IEEE/ACM 14th International Conference on Mining Software Repositories (MSR). IEEE; 2017. p. 425–435.

[6] Sebastio S, Baranov E, Biondi F, Decourbe O, Given-Wilson T, Legay A, et al. Optimizing symbolic execution for malware behavior classification. Computers & Security. 2020;93:101775. doi: 10.1016/j.cose.2020.101775

[7] Amer E, Zelinka I. A dynamic Windows malware detection and prediction method based on contextual understanding of API call sequence. Computers & Security. 2020;92:101760. doi: 10.1016/j.cose.2020.101760

[8] Huang W, Stokes JW. MtNet: a multi-task neural network for dynamic malware classification. In: International conference on detection of intrusions and malware, and vulnerability assessment. Springer; 2016. p. 399–418.

[9] Saxe J, Berlin K. Deep neural network based malware detection using two dimensional binary program features. In: 2015 10th International Conference on Malicious and Unwanted Software (MALWARE). IEEE; 2015. p. 11–20.

[10] Athiwaratkun B, Stokes JW. Malware classification with LSTM and GRU language models and a character-level CNN. In: 2017 IEEE International Conference on Acoustics, Speech and Signal Processing (ICASSP). IEEE; 2017. p. 2482–2486.

[11] Yakura H, Shinozaki S, Nishimura R, Oyama Y, Sakuma J. Neural malware analysis with attention mechanism. Computers & Security. 2019;87:101592. doi: 10.1016/j.cose.2019.101592

[12] Pascanu R, Stokes JW, Sanossian H, Marinescu M, Thomas A. Malware classification with recurrent networks. In: 2015 IEEE International Conference on Acoustics, Speech and Signal Processing (ICASSP). IEEE; 2015. p. 1916–1920.

[13] Tesauro GJ, Kephart JO, Sorkin GB. Neural networks for computer virus recognition. IEEE expert. 1996;11(4):5–6. doi: 10.1109/64.511768

[14] Chen H, Dean D, Wagner DA. Model Checking One Million Lines of C Code. In: NDSS. vol. 4; 2004. p. 171–185.

[15] Feng HH, Giffin JT, Huang Y, Jha S, Lee W, Miller BP. Formalizing sensitivity in static analysis for intrusion detection. In: IEEE Symposium on Security and Privacy, 2004. Proceedings. 2004. IEEE; 2004. p. 194–208.

[16] Ravi C, Manoharan R. Malware detection using windows api sequence and machine learning. International Journal of Computer Applications. 2012;43(17):12–16.

[17] Veeramani R, Rai N. Windows api based malware detection and framework analysis. In: International conference on networks and cyber security. vol. 25; 2012.

[18] Sornil O, Liangboonprakong C. Malware Classification Using N-grams Sequential Pattern Features. Journal of Information Processing and Management. 2013;4(5):59–67.

[19] Zhang Y, Sui Y, Pan S, Zheng Z, Ning B, Tsang I, et al. Familial clustering for weakly-labeled android malware using hybrid representation learning. IEEE Transactions on Information Forensics and Security. 2019;15:3401–3414. doi: 10.1109/TIFS.2019.2947861

[20] Forrest S, Hofmeyr SA, Somayaji A, Longstaff TA. A sense of self for unix processes. In: Proceedings 1996 IEEE Symposium on Security and Privacy. IEEE; 1996. p. 120–128.

[21] Lee W, Stolfo S. Data mining approaches for intrusion detection; 1998.

[22] Dahl GE, Stokes JW, Deng L, Yu D. Large-scale malware classification using random projections and neural networks. In: 2013 IEEE International Conference on Acoustics, Speech and Signal Processing. IEEE; 2013. p. 3422–3426.

[23] Bayer U, Comparetti PM, Hlauschek C, Kruegel C, Kirda E. Scalable, behavior-based malware clustering. In: NDSS. vol. 9. Citeseer; 2009. p. 8–11.

[24] BAYER U. TTAnalyze: A tool for analyzing malware. In: 15th Annual Conference of the European Institute for Computer Antivirus Research (EICAR), 2006; 2006.

[25] Agrawal R, Stokes JW, Selvaraj K, Marinescu M. Attention in recurrent neural networks for ransomware detection. In: ICASSP 2019-2019 IEEE International Conference on Acoustics, Speech and Signal Processing (ICASSP). IEEE; 2019. p. 3222–3226.

[26] Čeponis D, Goranin N. Evaluation of deep learning methods efficiency for malicious and benign system calls classification on the AWSCTD. Security and Communication Networks. 2019;2019.

[27] Čeponis D, Goranin N. Investigation of dual-flow deep learning models LSTM-FCN and GRU-FCN efficiency against single-flow CNN models for the host-based intrusion and malware detection task on univariate times series data. Applied Sciences. 2020;10(7):2373. doi: 10.3390/app10072373

[28] Damaševičius R, Venčkauskas A, Toldinas J, Grigaliūnas Š. Ensemble-Based Classification Using Neural Networks and Machine Learning Models for Windows PE Malware Detection. Electronics. 2021;10(4):485. doi: 10.3390/electronics10040485

[29] Qiu J, Zhang J, Luo W, Pan L, Nepal S, Wang Y, et al. A3CM: automatic capability annotation for android malware. IEEE Access. 2019;7:147156–147168. doi: 10.1109/ACCESS.2019.2946392

[30] Huang YT, Chen YY, Yang CC, Sun Y, Hsiao SW, Chen MC. Tagging Malware Intentions by Using Attention-Based Sequence-to-Sequence Neural Network. In: Australasian Conference on Information Security and Privacy. Springer; 2019. p. 660–668.

[31] CARO. A New Virus Naming Convention;. http://www.caro.org/articles/naming.html.

[32] Beck D, Connolly J. The common malware enumeration initiative. In: Proceedings of the Virus Bulletin Conference; 2006.

[33] Hsiao SW, Sun YS, Chen MC. Hardware-Assisted MMU Redirection for In-Guest Monitoring and API Profiling. IEEE Transactions on Information Forensics and Security. 2020;15:2402–2416. doi: 10.1109/TIFS.2020.2969514

[34] Sutskever I, Vinyals O, Le QV. Sequence to Sequence Learning with Neural Networks. Advances in Neural Information Processing Systems. 2014;27:3104–3112.

[35] Luong MT, Pham H, Manning CD. Effective Approaches to Attention-based Neural Machine Translation. In: Proceedings of the 2015 Conference on Empirical Methods in Natural Language Processing; 2015. p. 1412–1421.

[36] Kim S, Hori T, Watanabe S. Joint CTC-attention based end-to-end speech recognition using multi-task learning. In: 2017 IEEE international conference on acoustics, speech and signal processing (ICASSP). IEEE; 2017. p. 4835–4839.

[37] Badrinarayanan V, Kendall A, Cipolla R. Segnet: A deep convolutional encoder-decoder architecture for image segmentation. IEEE transactions on pattern analysis and machine intelligence. 2017;39(12):2481–2495. doi: 10.1109/TPAMI.2016.2644615 28060704

[38] Chen LC, Zhu Y, Papandreou G, Schroff F, Adam H. Encoder-decoder with atrous separable convolution for semantic image segmentation. In: Proceedings of the European conference on computer vision (ECCV); 2018. p. 801–818.

[39] Hochreiter S, Schmidhuber J. Long short-term memory. Neural computation. 1997;9(8):1735–1780. doi: 10.1162/neco.1997.9.8.1735 9377276

[40] Zhou B, Khosla A, Lapedriza A, Oliva A, Torralba A. Learning deep features for discriminative localization. In: Proceedings of the IEEE conference on computer vision and pattern recognition; 2016. p. 2921–2929.

[41] Chorowski J, Bahdanau D, Cho K, Bengio Y. End-to-end continuous speech recognition using attention-based recurrent nn: First results. In: NIPS 2014 Workshop on Deep Learning, December 2014; 2014.

[42] Vaswani A, Shazeer N, Parmar N, Uszkoreit J, Jones L, Gomez AN, et al. Attention is All you Need. In: NIPS; 2017.

[43] Xu K, Ba J, Kiros R, Cho K, Courville A, Salakhudinov R, et al. Show, attend and tell: Neural image caption generation with visual attention. In: International conference on machine learning. PMLR; 2015. p. 2048–2057.

[44] Kingma DP, Ba J. Adam: A method for stochastic optimization. arXiv preprint arXiv:14126980. 2014.

[45] Pedregosa F, Varoquaux G, Gramfort A, Michel V, Thirion B, Grisel O, et al. Scikit-learn: Machine Learning in Python. Journal of Machine Learning Research. 2011;12:2825–2830.

[46] Sethi K, Kumar R, Sethi L, Bera P, Patra PK. A novel machine learning based malware detection and classification framework. In: 2019 International Conference on Cyber Security and Protection of Digital Services (Cyber Security). IEEE; 2019. p. 1–4.

[47] Stiawan D, Arifin MAS, Idris MY, Budiarto R, et al. IoT Botnet Malware Classification Using Weka Tool and Scikit-learn Machine Learning. In: 2020 7th International Conference on Electrical Engineering, Computer Sciences and Informatics (EECSI). IEEE; 2020. p. 15–20.

[48] Jolliffe I. Principal component analysis. Technometrics. 2003;45(3):276. doi: 10.1198/tech.2003.s783

[49] Van der Maaten L, Hinton G. Visualizing data using t-SNE. Journal of machine learning research. 2008;9(11).

